# Supplementary material for: Global emergence and population dynamics of divergent serotype 3 CC180 pneumococci
Source: PLoS Pathog. 2018 Nov 26;14(11):e1007438. doi: 10.1371/journal.ppat.1007438 (PMC6283594; doi:10.1371/journal.ppat.1007438)
Supplement: S1 Methods — (DOCX) [file ppat.1007438.s001.docx]

**Supplemental Methods**

*Coalescent analysis*

For major Clade I (hereon referred to as Clade I-α) and Clade II, coalescent analysis was performed using BEAST v1.8.4 [1]. For each clade, a combination of strict and relaxed molecular clock models and constant, exponential, and Gaussian Markov random field (GMRF) demographic models were tested using recombination-free SNP alignments, HKY nucleotide substitution model, and ascertainment bias correction [3,4]. Markov chain Monte Carlo (MCMC) chain lengths for each model were 500 million and 100 million for Clade I-α and II, respectively. MCMC chains were sampled to obtain 10,000 trees and 10,000 parameter estimates in the posterior distribution, and effective sampling size (ESS) values were assessed to determine sufficient mixing using Tracer v1.6.0. Marginal likelihood estimates (MLE) were obtained for each model using path-sampling and stepping-stone analysis, and models were compared using Bayes Factors [5,6]. To confirm temporal signal, date randomization tests using 10 replicates were performed and evolutionary rate estimates were compared for Clades I-α and II [34,35]. If the mean evolutionary rate estimated with the correct sampling times is not contained within any of the 95% credible intervals of those estimated from the date-randomized data sets then there is sufficient temporal signal. Finally, parameter estimates for the evolutionary rate, root height, and *N_e_* were obtained from the best-fit model and compared between clades.

*Opsonophagocytic killing assay (OPKA)*

The human cell line HL-60 (promyelocytic leukemia cells; American Type Culture Collection, Cat. No. CCL-240) was used as a source of effector cells. HL-60 cells were maintained in Iscove’s Modified Dulbecco’s Medium (IMDM) (Corning, Cat. No. 10-016-CV) containing 2 mM L-glutamine, 25 mM HEPES and supplemented with 20% fetal bovine serum (American Type Culture Collection, Cat. No. 30-2020). Cells were grown in suspension to ≤ 1x10^6^ cells/ml at 37ºC in 5% CO_2_. Differentiation was carried out in supplemented IMDM (as described above) containing 0.8% *N,N*-dimethylformamide (Sigma, Cat. No. D4551), using inoculation of 5x10^5^ cells/ml as determined by viable counts with trypan blue exclusion. Cultures were incubated for 5 days without replacing differentiation medium.

For the functional assay, bacteria, differentiated HL-60 cells, serum and complement source were prepared as described below. For preparation of the serum, each serum sample was diluted in opsonophagocytosis buffer for a total of five dilutions. Once all serum samples were prepared, 80 µl of bacterial suspension appropriately diluted (~1000 CFU) was added to each well. Following a 30-minute incubation at 37ºC, 10 µl baby rabbit complement (Pel-Freez, Cat. No. 31064) was added to each well. The assay plate was incubated for another 30 minutes at 37ºC. Immediately after this, differentiated HL-60 cells were added to each well at an effector/target cell ratio of 400/1. For each assay, differentiated cells were harvested by centrifugation (1700 rpm) for 10 min at room temperature. The cell pellet was washed twice in Hanks’ buffer without Ca^2+^ and Mg^2+^ (Corning, Cat. No. 21-022-CV). Finally, the cells were resuspended to 1x10^7^ cells/ml in Hanks’ buffer with Ca^2+^ and Mg^2+^ (Corning, Cat. No. 21-023-CV) supplemented with 0.1% gelatin. The total number of HL-60 cells to be added per well was 4x10^5^ in a 40 µl volume. The assay plate was incubated at 37ºC for 45 minutes with horizontal shaking (700 rpm) to promote the phagocytic process. All samples were run in triplicate. An aliquot from each well was plated onto solid medium (trypticase soy agar with 5% sheep blood) to enumerate viable colony forming units (CFU) after overnight incubation. A viable count of the initial number of bacteria added per well at time zero was included in each run. For each sample, the CFU counts at time point 45 minutes was divided by the CFU counts at time point 0 to determine the percentage of CFU that survived.

References

1. Drummond AJ, Suchard M a, Xie D, Rambaut A. Bayesian phylogenetics with BEAUti and the BEAST 1.7. Mol Biol Evol. 2012; 1–5. doi:10.1093/molbev/mss075

2. Hall MD, Woolhouse MEJ, Rambaut A. The effects of sampling strategy on the quality of reconstruction of viral population dynamics using Bayesian skyline family coalescent methods: A simulation study. Virus Evol. 2016;2: vew003. doi:10.1093/ve/vew003

3. Gray RR, Tatem AJ, Johnson J a, Alekseyenko A V, Pybus OG, Suchard M a, et al. Testing spatiotemporal hypothesis of bacterial evolution using methicillin-resistant Staphylococcus aureus ST239 genome-wide data within a bayesian framework. Mol Biol Evol. 2011;28: 1593–603. doi:10.1093/molbev/msq319

4. Harris SR, Feil EJ, Holden MTG, Quail M a, Nickerson EK, Chantratita N, et al. Evolution of MRSA during hospital transmission and intercontinental spread. Science. 2010;327: 469–74. doi:10.1126/science.1182395

5. Baele G, Lemey P, Vansteelandt S. Make the most of your samples: Bayes factor estimators for high-dimensional models of sequence evolution. BMC Bioinformatics. 2013;14: 85. doi:10.1186/1471-2105-14-85

6. Kass R. Bayes factors. J Am Stat Assoc. 1995;90: 773–795.

7. Duchêne S, Duchêne D, Holmes EC, Ho SYW. The Performance of the Date-Randomization Test in Phylogenetic Analyses of Time-Structured Virus Data. Mol Biol Evol. Oxford University Press; 2015;32: 1895–1906. doi:10.1093/molbev/msv056
