# Supplementary figures and images for: Global emergence and population dynamics of divergent serotype 3 CC180 pneumococci
Source: PLoS Pathog. 2018 Nov 26;14(11):e1007438. doi: 10.1371/journal.ppat.1007438 (PMC6283594; doi:10.1371/journal.ppat.1007438)

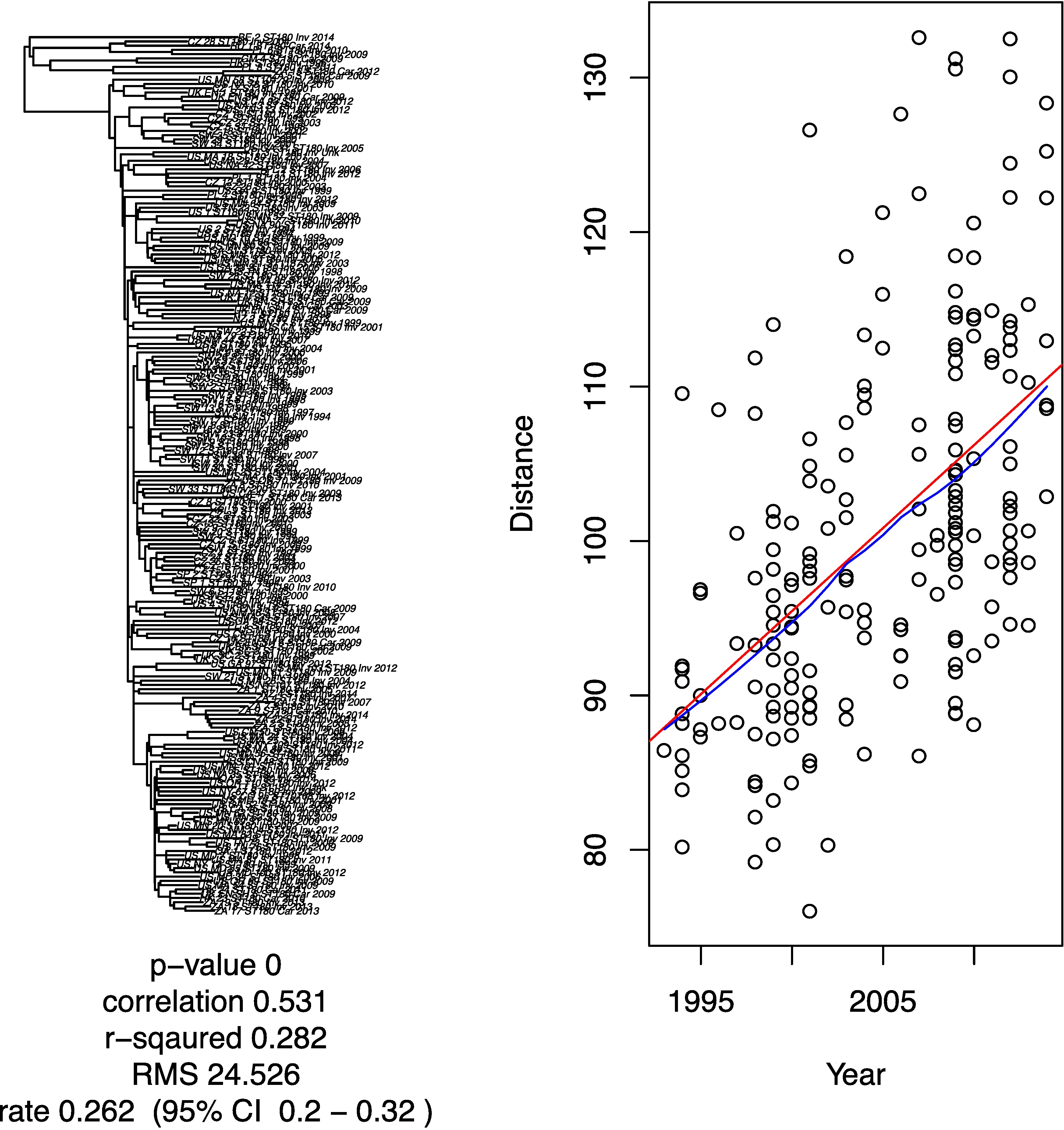

Supplement: S1 Fig — The tip dates, assigned using the collection date of the isolate, is regressed upon the distance from the estimated root in the maximum likelihood phylogeny to determine temporal signal. (TIF) [file ppat.1007438.s003.tif]

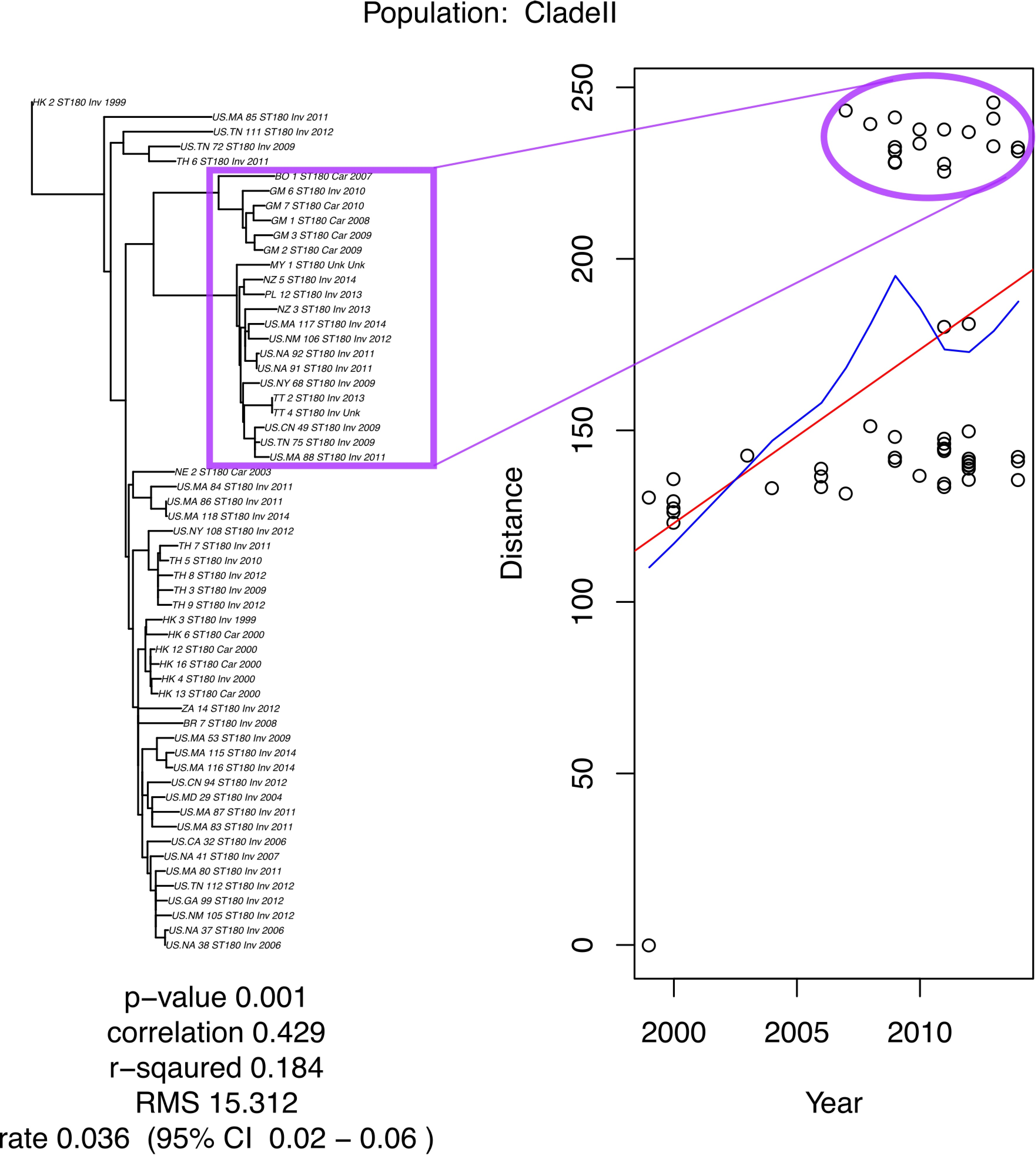

Supplement: S2 Fig — The tip dates, assigned using the collection date of the isolate, is regressed upon the distance from the estimated root in the maximum likelihood phylogeny to determine temporal signal. The corresponding taxa for the points in the upper-right of the regression plot (indicated with a purple oval) are identified on the phylogeny to the left. The root-to-tip correlation was also independently assessed for the sub-clade indicated in purple and found to have a significant correlation. (TIF) [file ppat.1007438.s004.tif]

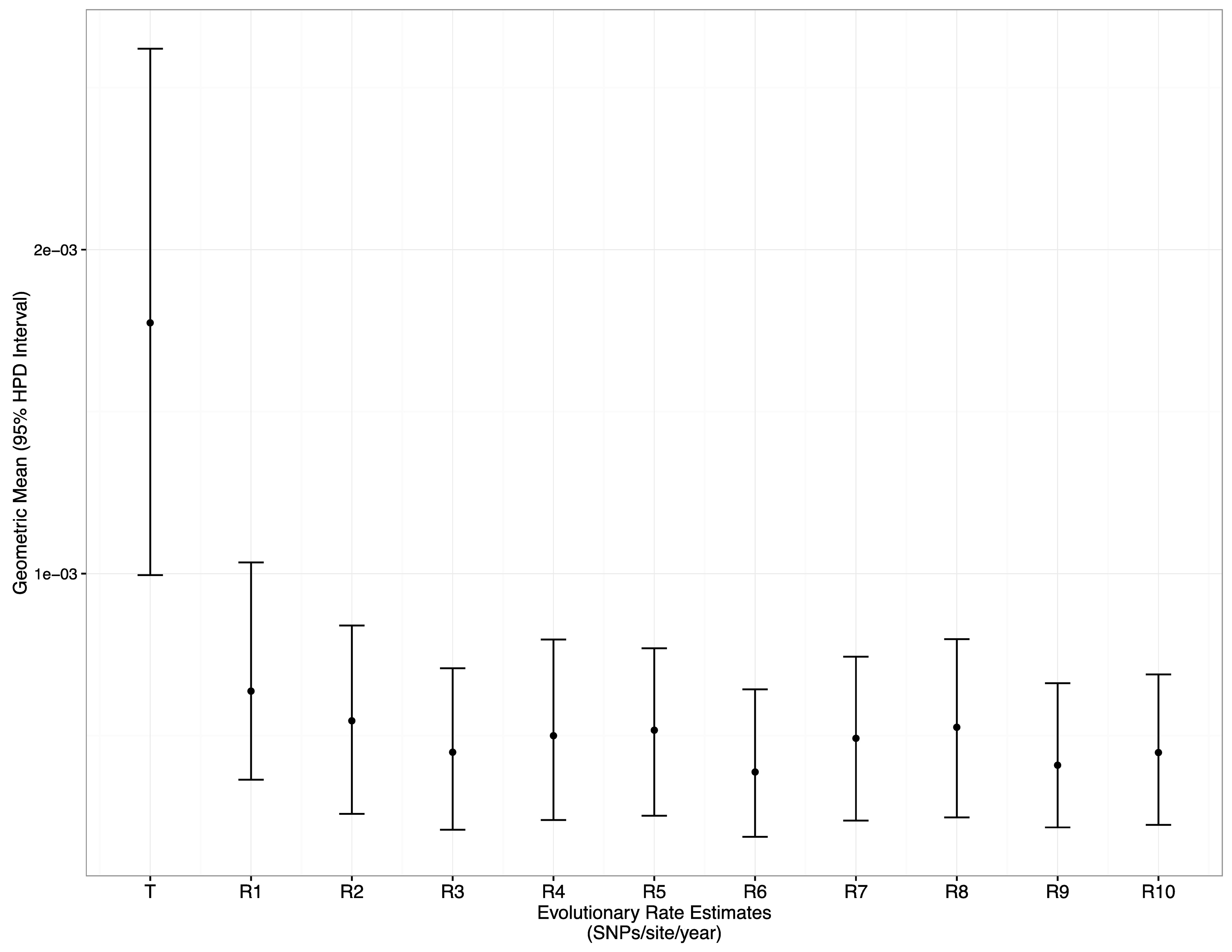

Supplement: S3 Fig — The estimated evolutionary rate for the BEAST run with correct date assignment (T:true) appears at the far left of the x-axis, with the realizations for 10 date permutation runs to the right. Evolutionary rates in this figure are not corrected for alignment length. (TIF) [file ppat.1007438.s005.tif]

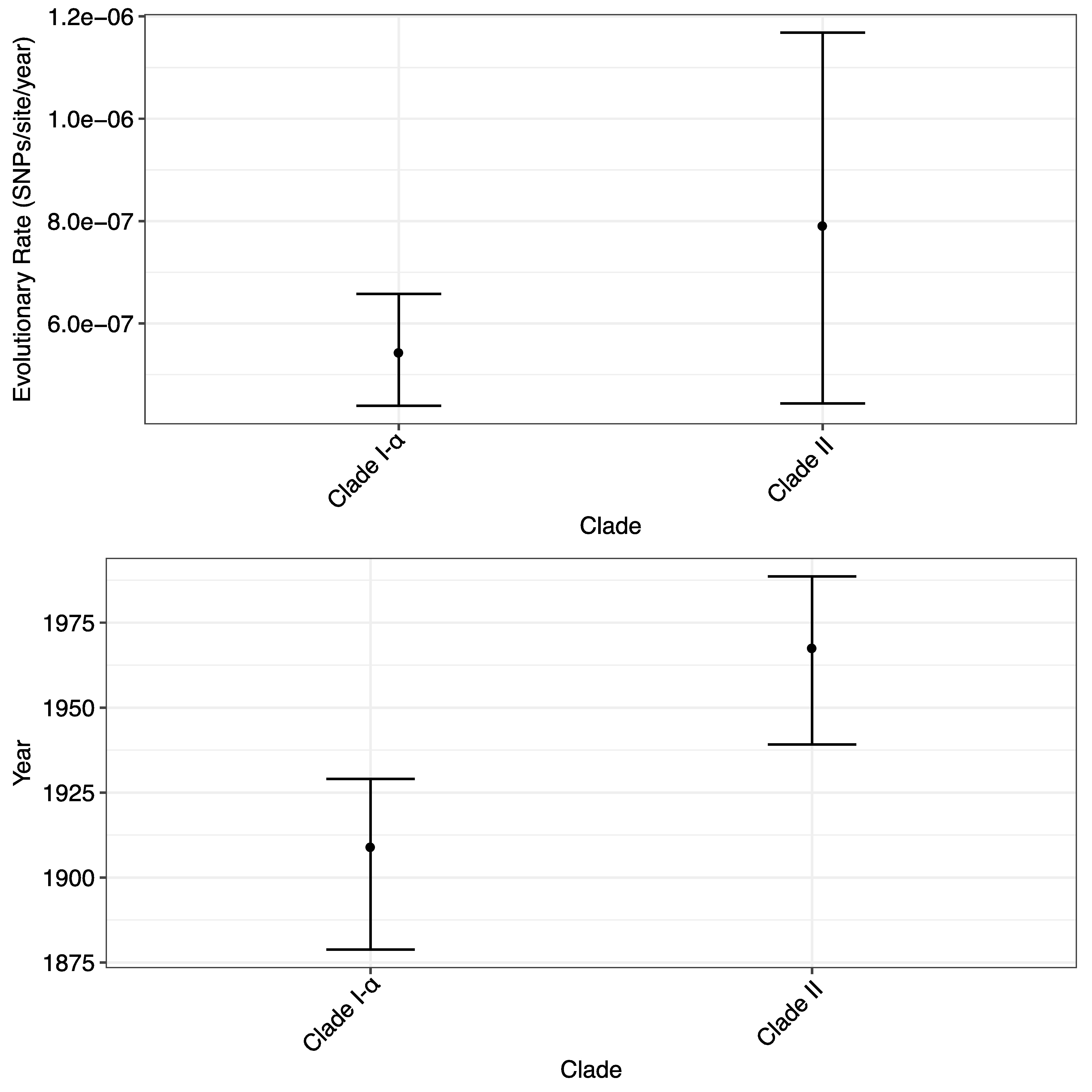

Supplement: S4 Fig — Evolutionary rate is scaled in SNPs/site/year and TMRCA as the year of the estimated root height. Clade I is significantly “older” then the recently emerged Clade II. (TIF) [file ppat.1007438.s006.tif]

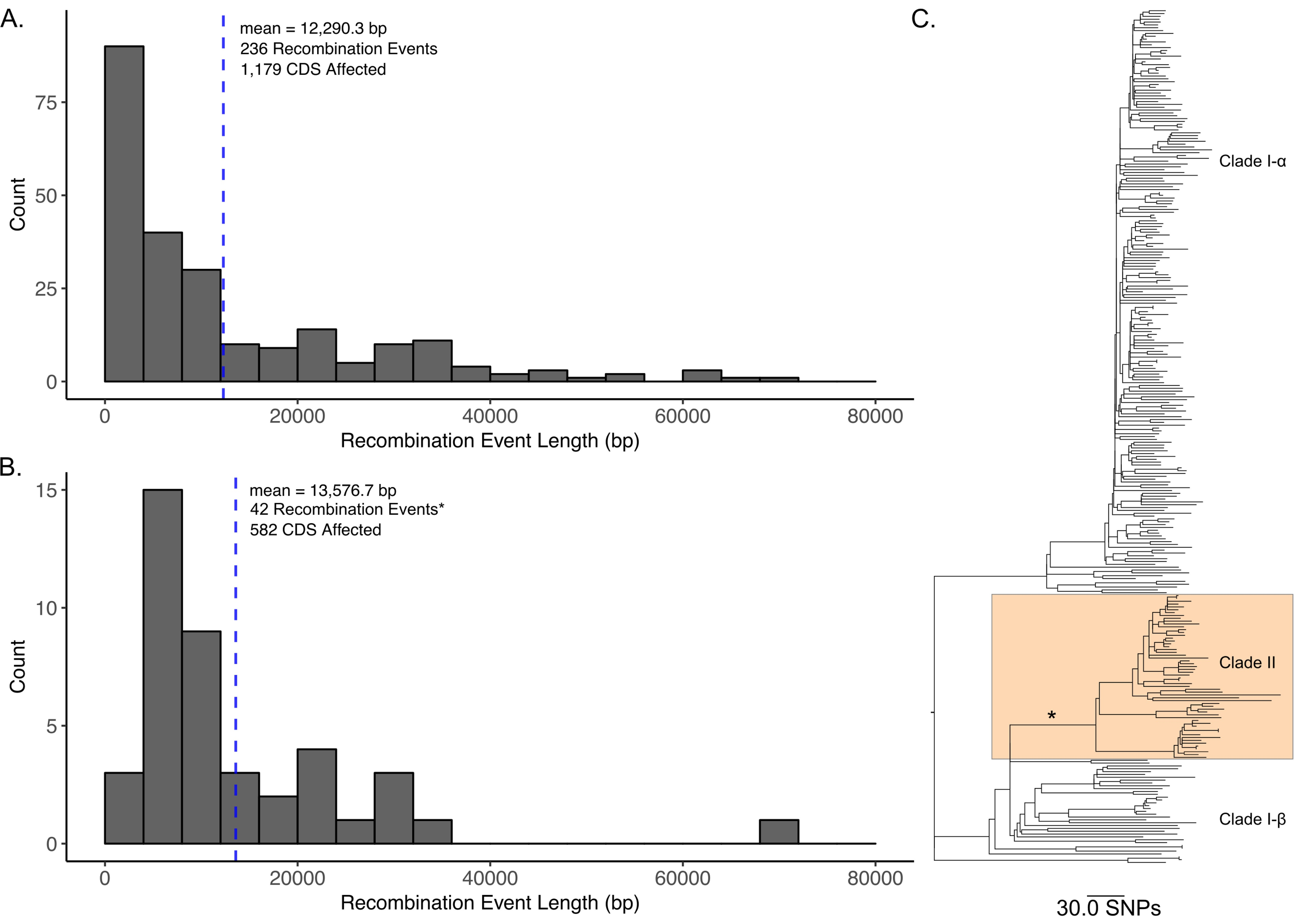

Supplement: S5 Fig — A.) Distribution of inferred recombination block sizes for the entire sample and B.) the major branch leading to Clade II (shaded and denoted with an asterisk). C.) Phylogeny depicting the population structure of CC180 with Clade II shaded. In total, 236 Recombination events impacted 1,179 coding sequences (CDS), with 42 of those events occurring on the major branch leading to Clade II. The largest event, a 68,164 bp recombination block spanning the region 407,458–475,621 on the OXC141 reference genome, impacting 70 CDS and 13 repeat regions. Pathways including these CDSs involve metabolism (n = 17), genetic information processing (n = 10), signaling (n = 7), and environmental information processing (n = 5). Notably, rpoE recombination and DNA repair protein (SPNOXC_04590) is included in this region. (TIF) [file ppat.1007438.s007.tif]

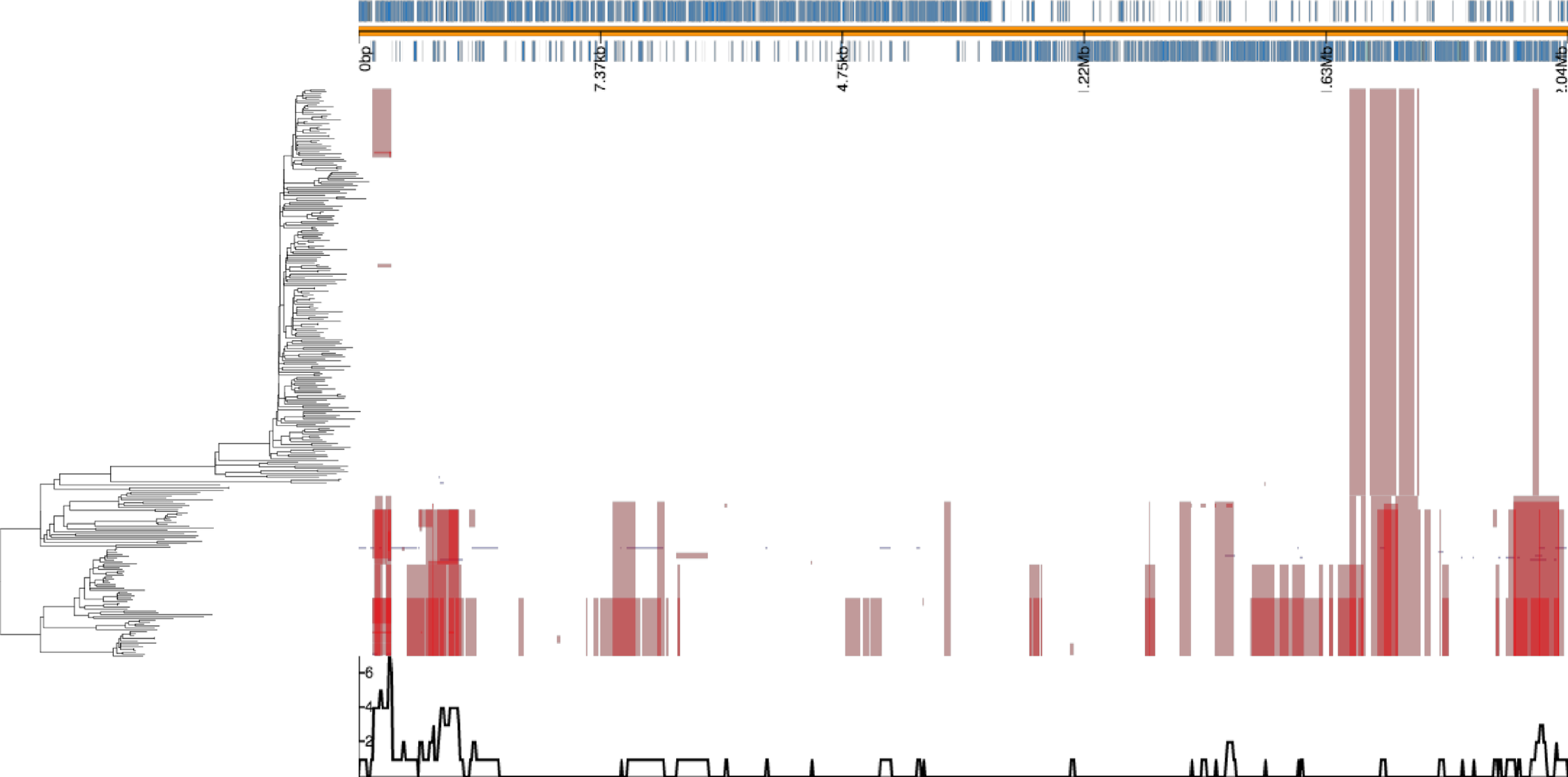

Supplement: S6 Fig — The left side of the figure represents a maximum likelihood phylogeny of CC180 isolates inferred from a recombination-censored alignment using Gubbins. The tree has been out-group rooted as described in the methods and clades are ordered Clade I-α, I-β, and II, from top to bottom. On the top of the figure is a linear depiction of the 2,036,867 bp S. pneumoniae OXC141 reference genome on which recombination events are mapped. The bottom ribbon is a graph of recombination events distributed linearly across the genome. The center panel indicated recombination events among taxa. Red blocks display events occurring on ancestral branches and are therefore shared by a group of taxa. Darker shades of red indicate multiple events have occurred in a genomic region. Blue blocks signify recombination events that occurred on terminal branches and are unique to only one isolate. Output of Gubbins was visualized in Phandango, which was used to construct this figure. (TIF) [file ppat.1007438.s008.tif]

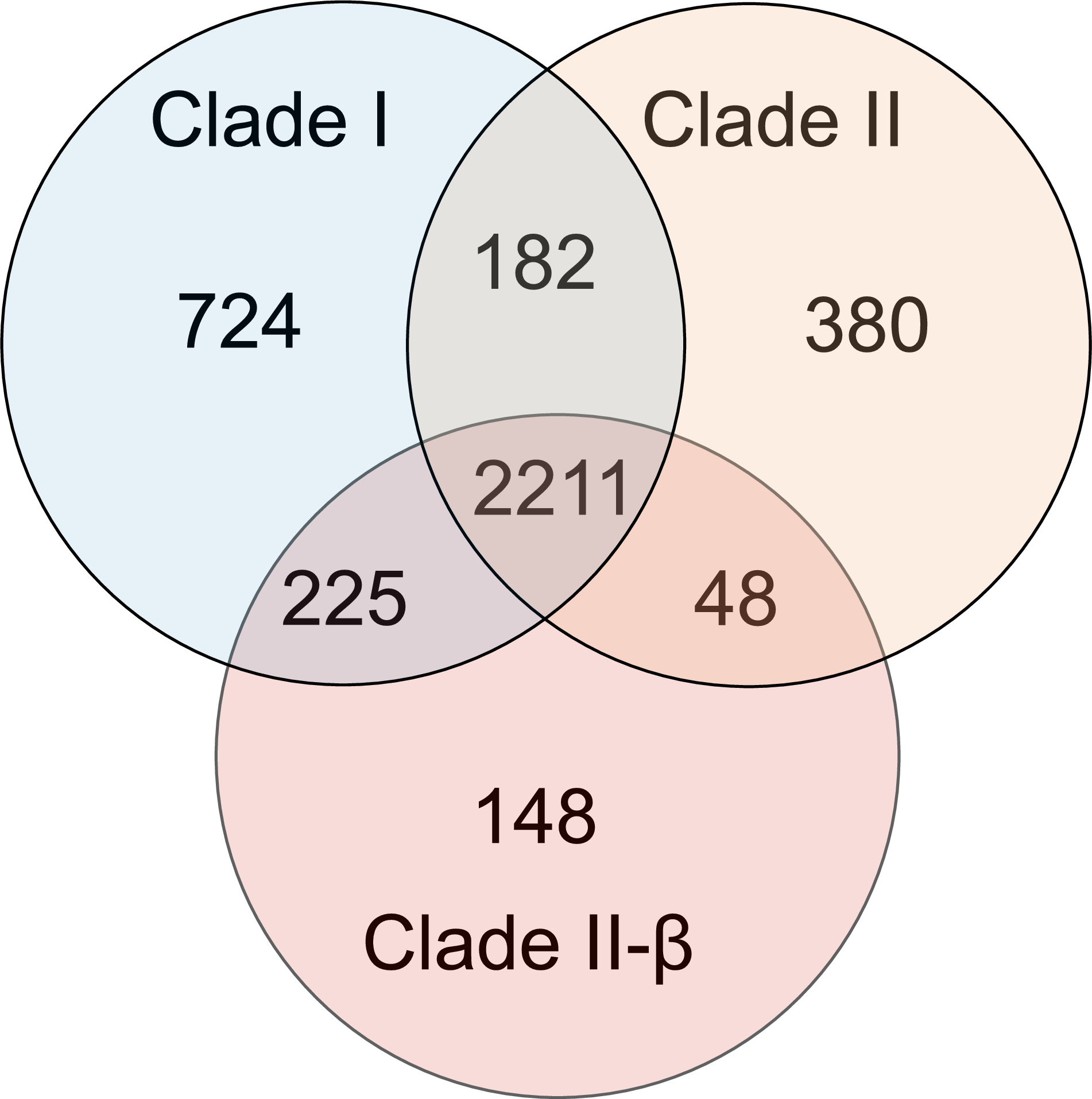

Supplement: S7 Fig — COGs possessed by at least one individual within a clade were counted toward the “gene-pool” for each comparison. (TIF) [file ppat.1007438.s009.tif]

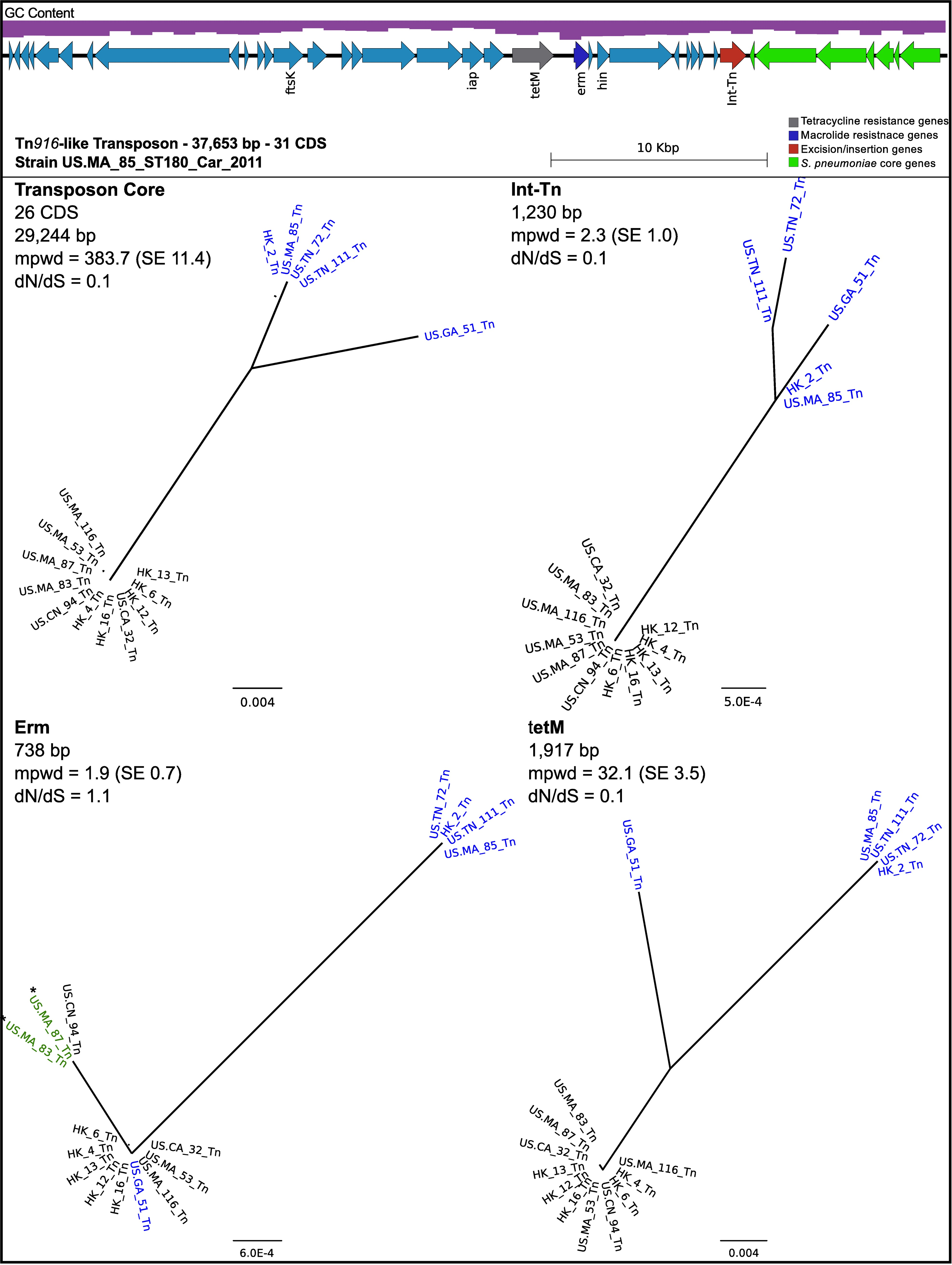

Supplement: S8 Fig — The core genome phylogeny and genealogies are labeled with length of alignment, mean pairwise difference (mpwd), and the ratio of non-synonymous to synonymous mutations (dN/dS). The phylogenies show a clear delineation between two distinct transposons (blue and black tip labels). In the ermB genealogy, two isolates that are macrolide susceptible are colored in green. (TIF) [file ppat.1007438.s010.tif]

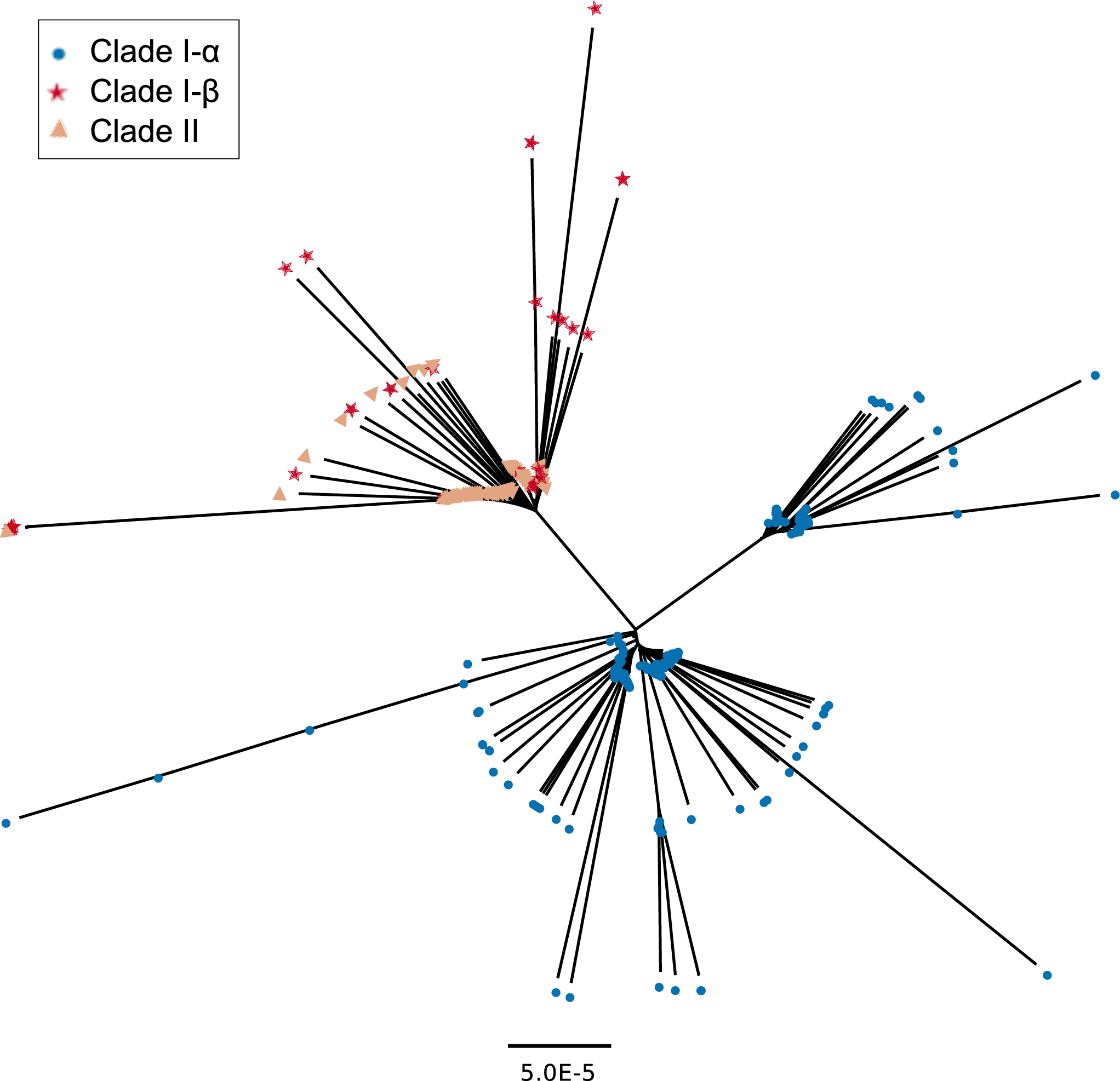

Supplement: S9 Fig — Tips are labeled and colored by clade. Despite the clear divergence of Clades I-α and II+I-β, very little nucleotide diversity exists. (TIF) [file ppat.1007438.s011.tif]

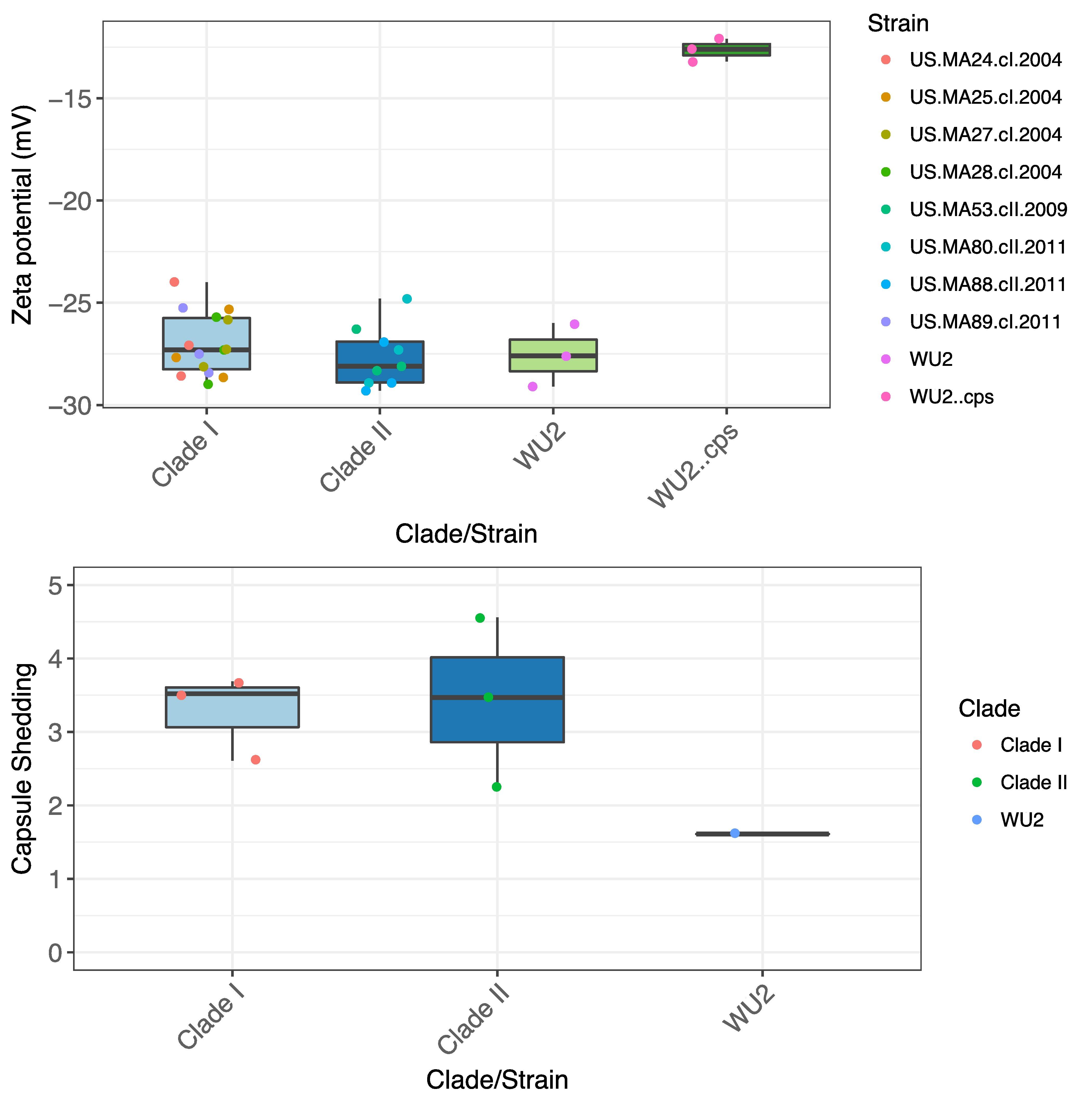

Supplement: S10 Fig — ST3 Serotype 3 strain WU2 is used as a control for both experiments and WU2ΔCPS is included in the analysis of zeta potentials. (TIF) [file ppat.1007438.s012.tif]

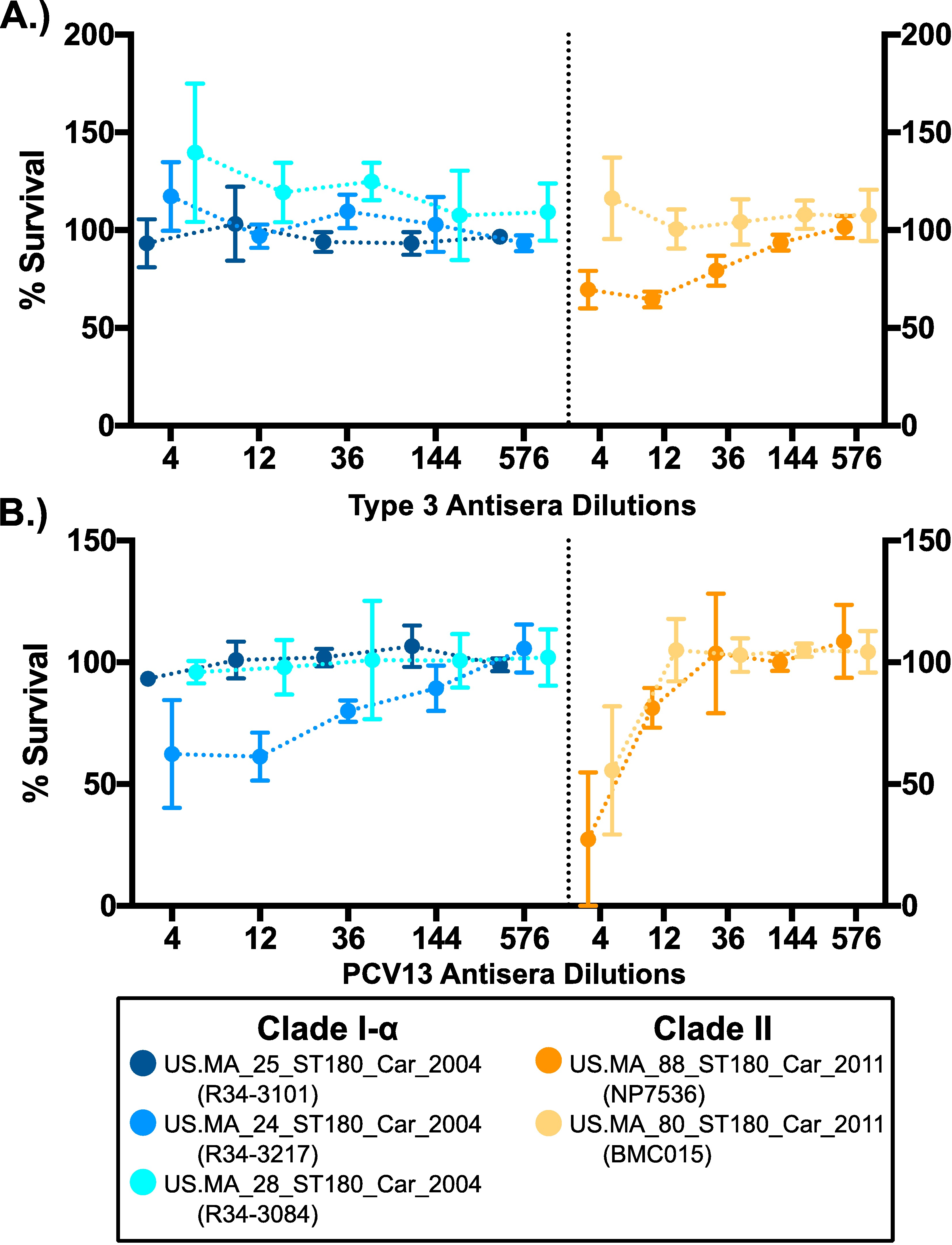

Supplement: S11 Fig — Antisera dilutions are labeled on the x-axis and percent survival on the y-axis. Clade I-α isolates are on the left half of the x-axis and Clade II appear on the right. Opsonophagocytic killing appears as a reduction in percent survival with increasing concentrations of antisera. (TIF) [file ppat.1007438.s013.tif]
