# Supplementary material for: Global emergence and population dynamics of divergent serotype 3 CC180 pneumococci
Source: PLoS Pathog. 2018 Nov 26;14(11):e1007438. doi: 10.1371/journal.ppat.1007438 (PMC6283594; doi:10.1371/journal.ppat.1007438)
Supplement: S1 Table — For each clade, strict (SC) and relaxed (RC) molecular clock models were compared for constant, exponential, and GMRF Skygrid demographic models. Log marginal likelihood estimates (MLE) from path-sampling (PS) and stepping-stone (SS) analysis were used to calculate Bayes Factors for model comparison. Log Bayes Factors (BF) are specified for each molecular clock and demographic model comparison. The date of the most recent common ancestor (TMRCA) and evolutionary rate, scaled in SNPs/site/year are presented for the final model with corresponding highest posterior densities (HPD). (DOCX) [file ppat.1007438.s014.docx]

| **Clade** | **Constant (Con)**  **Demographic Model** | | | **Exponential (Exp)**  **Demographic Model** | | | **GMRF Skygrid (Sky)**  **Demographic Model** | | | **Demography** | | **TMRCA [mean**  **(95% HPD)]** | **Evolutionary Rate (SNPs/site/yr)**  **[mean (95% HPD)]** |
| --- | --- | --- | --- | --- | --- | --- | --- | --- | --- | --- | --- | --- | --- |
|  | **Molecular Clock**  **Model** | |  | **Molecular Clock Model** | |  | **Molecular Clock Model** | |  | **Exp v Sky** | **Con v Sky** |  |  |
|  | **SC**  **(log MLE)** | **RC**  **(log MLE)** | **Log BF**  **Clock** | **SC**  **(log MLE)** | **RC**  **(log MLE)** | **Log BF**  **Clock** | **SC**  **(log MLE)** | **RC**  **(log MLE)** | **Log BF**  **Clock** | **Log**  **BF** | **Log**  **BF** |  |  |
| **I-α** |  |  |  |  |  |  |  |  |  |  |  | 1909 [1878,1929] | 5.4x10-7  [6.6x10-7, 4.4x10-7] |
| PS | -36063.7 | -36066.0 | -2.3 | -35909.4 | -35897.1 | 12.2 | -35932.5 | -35643.7 | 288.8 | 253.4 | 11.8 |  |  |
| SS | -36066.0 | -36067.4 | -1.5 | -35910.5 | -35899.7 | 10.8 | -35933.0 | -35646.2 | 286.8 | 253.5 | 11.4 |  |  |
| **II** |  |  |  |  |  |  |  |  |  |  |  | 1968  [1939, 1989] | 7.9x10-7  [1.2x10-6, 4.4x10-7] |
| PS | -6926.3 | -6916.0 | 10.4 | -6888.7 | -6884.1 | 4.6 | -6914.2 | -6904.2 | 10.0 | -20.1 | 93.4 |  |  |
| SS | -6926.2 | -6915.9 | 10.4 | -6889.0 | -6884.2 | 4.8 | -6915.3 | -6904.5 | 10.8 | -20.3 | 95.6 |  |  |
