# Supplementary material for: Global emergence and population dynamics of divergent serotype 3 CC180 pneumococci
Source: PLoS Pathog. 2018 Nov 26;14(11):e1007438. doi: 10.1371/journal.ppat.1007438 (PMC6283594; doi:10.1371/journal.ppat.1007438)
Supplement: S2 Table — 2-0-111 possesses first step PBP-2X 111 allele that is associated with a slightly elevated penicillin MIC of 0.06. All other profiles are associated with a penicillin MIC (μg/ml) ≤0.03. (DOCX) [file ppat.1007438.s015.docx]

| **PBP Profile** | **Clade I-α** | | **Clade II** | | **Clade I-β** | | **Total** | **Percent** |
| --- | --- | --- | --- | --- | --- | --- | --- | --- |
|  | *Count* | *Percent* | *Count* | *Percent* | *Count* | *Percent* |  |  |
| 2-0-111 | 0 | 0.0% | 4 | 9.3% | 0 | 0.0% | 4 | 1.5% |
| 2-0-136 | 0 | 0.0% | 2 | 4.7% | 0 | 0.0% | 2 | 0.7% |
| 2-0-2 | 10 | 5.3% | 37 | 86.0% | 36 | 97.3% | 83 | 30.7% |
| 2-3-2 | 165 | 86.8% | 0 | 0.0% | 0 | 0.0% | 165 | 61.1% |
| 2-3-285 | 1 | 0.5% | 0 | 0.0% | 0 | 0.0% | 1 | 0.4% |
| 2-3-39 | 1 | 0.5% | 0 | 0.0% | 0 | 0.0% | 1 | 0.4% |
| 2-96-2 | 1 | 0.5% | 0 | 0.0% | 0 | 0.0% | 1 | 0.4% |
| 2-NF-2 | 12 | 6.3% | 0 | 0.0% | 1 | 2.7% | 13 | 4.8% |
| *Total* | *190* |  | *43* |  | *37* |  | *270* |  |
